# Selective Removal of Mo and W from Acidic Leachates Using Thiourea Modified Macroporous Anion Exchanger

**DOI:** 10.3390/molecules30183803

**Published:** 2025-09-18

**Authors:** Akmaral Ismailova, Dilyara Rashit, Tomiris Kossova, Yerbol Tileuberdi

**Affiliations:** 1 Faculty of Chemistry and Chemical Technology, Al-Farabi Kazakh National University, 71 Al-Farabi Avenue, 050040 Almaty, Kazakhstan; 2 Faculty of Natural Sciences and Geography, Abai Kazakh National Pedagogical University, 13 Dostyk Avenue, 050010 Almaty, Kazakhstan; er.tileuberdi@gmail.com

**Keywords:** molybdenum, tungsten, selective sorption, modification, sorption kinetics, anion-exchange resin

## Abstract

In this study, a commercial anion-exchange resin (D301), known for high regenerability but limited selectivity, was chemically modified to enhance its sorption performance. The modification included graft polymerization of glycidyl methacrylate followed by thiourea functionalization, yielding a new sorbent, TD301, with chelating functional groups. Characterization using SEM/EDS, IR spectroscopy, XPS, and zeta potential measurements confirmed the successful introduction of sulfur- and nitrogen-containing groups, increased surface roughness, and decreased surface charge in the pH range 2–6. These changes shifted the sorption mechanism from nonspecific ion exchange to selective coordination. Sorption properties of TD301 were evaluated in mono- and bimetallic Mo–W systems, as well as in solutions obtained from real ore decomposition. The modified sorbent showed fast sorption kinetics and high selectivity for Mo(VI) at pH 1.5, while retaining high W(VI) uptake at pH 0.5. In binary systems, separation factors (*α*) reached 128.4, greatly exceeding those of unmodified D301. In real leachates (Mo ≈ W ≈ 0.04 g/L), TD301 selectively extracted W at pH 0.66 and Mo at pH 1.5. These findings demonstrate that TD301 is an effective sorbent for pH-dependent Mo/W separation in complex matrices, with potential for resource recovery, wastewater treatment, monitoring, and suitability for repeated use.

## 1. Introduction

Molybdenum (Mo) and tungsten (W) are considered strategically important metals, widely used in producing high-strength alloys, catalysts, and electronic components because of their exceptional thermal and chemical stability properties [1,2], which also justify their increasing use in medical applications [3,4,5,6,7]. Although molybdenum and tungsten are often regarded as relatively low-toxicity metals, recent studies have highlighted their potential risks to human health and the environment, particularly at elevated concentrations. Molybdenum can cause problems with metabolism [8] and reproduction [9], while tungsten is linked to DNA damage [10], stress on cells [11], and buildup in living things. Their persistent anionic forms in aqueous environments, resistance to biodegradation, and increasing industrial use raise concerns regarding their emergence as new environmental pollutants. The World Health Organization recommends a molybdenum concentration limit of 70 µg/L in drinking water [12], whereas proposed screening levels for tungsten range from 100 to 200 µg/L [13].

Effective separation and recovery of these metals is essential not only to ensure the quality of final products and optimize industrial processes but also to prevent their uncontrolled release into the environment. As the main sources of metals run out and the amount of metal in raw materials decreases, more people are interested in using secondary sources—like industrial wastewater, used catalysts, and leftover materials—not just as alternative ways to obtain valuable metals but also as opportunities to clean up the environment and reduce pollution [14]. Nonetheless, the limited efficiency of conventional separation methods often results in considerable losses of valuable metals and limits the purity of the final products [15,16,17].

Various methods have been employed for the extraction and separation of Mo and W, including precipitation, solvent extraction [14,18,19,20,21,22], membrane technologies, and sorption processes [4,23,24]. Among these, sorption techniques, particularly those utilizing anion-exchange resins [15,16,17,25,26,27] have attracted significant attention due to their high selectivity, low energy consumption, and environmental safety [28,29]. Macroporous anion-exchange resins, like D301 and its modified versions, are very effective at selectively capturing molybdenum and tungsten oxoanions from water solutions [16].

Nevertheless, the separation of Mo and W remains a challenging task due to their chemical similarities and analogous behavior in solution. In neutral and mildly alkaline conditions (pH 6–9), Mo and W predominantly exist as MoO_4_^2−^ and WO_4_^2−^ oxoanions or as polymetallic species such as Mo_7_O_24_^6−^ and W_7_O_24_^6−^ [1,2,3,22]. As the pH decreases, molybdenum and tungsten exist primarily as protonated species (e.g., HMoO_4_^−^, HWO_4_^−^) and hydrated oxoacids (H_2_MoO_4_, H_2_WO_4_). Under strongly acidic conditions (pH < 2), these metals tend to form large polyoxometalates such as Mo_8_O_26_^4−^ and W_12_O_40_^8−^ [28,30,31]. At pH < 0.5, molybdenum exists predominantly in cationic form, while tungsten is present as tungstic acid. These changes in the chemical forms of the metals affect their electrical charge and arrangement, making it harder for them to interact specifically with the functional groups of sorbents.

Acidic media are characteristic of many real-world technological streams, including acid leaching processes of secondary raw materials [14,20,21], which demonstrate the importance of sorbents that are both stable and effective under such conditions. However, it has previously been shown that sorption efficiency for Mo and W declines markedly in strongly acidic solutions due to the weakened interaction between bulky polyoxoanions and conventional anion-exchange groups [32,33].

To enhance sorbent performance, various strategies for resin modification have been proposed, including the introduction of tertiary amine groups and chelating fragments containing phosphorus or sulfur [17,25,26,27,28], which improve selectivity and chemical resistance in aggressive environments. Even though they could create strong connections with Mo(VI) using sulfur and nitrogen atoms, thiourea-functionalized derivatives have not been researched enough [16].

Thiourea-functionalized resins have been applied for the removal of other metals successfully. As demonstrated by the study, a urea/thiourea-modified D301 resin efficiently removed Cd(II), Hg(II), and Pb(II) ions from aqueous solutions [26]. According to another investigation, D301 modified with N- and S-containing groups showed selective adsorption of AuCl_4_^−^ [27]. These findings show the adaptability of thiourea functionalization and support its further application in the selective separation of molybdenum and tungsten.

Because of these factors, creating new macroporous anion exchangers with thiourea is very important, as these materials are expected to effectively capture molybdenum in acidic solutions, even when there is a lot of tungsten present.

The study focused on developing and explaining a new sorbent called TD301, made from D301 and treated with thiourea to effectively capture Mo(VI) while avoiding W(VI) in acidic water. This sorbent is intended for application in the treatment of industrial wastewater and the mitigation of environmental contamination by critical raw elements.

## 2. Results and Discussions

### 2.1. Comparative Analysis of Physicochemical Properties of Sorbents

Understanding the detailed physical and chemical properties of the sorbents is important for knowing how they are built, how they work, and how well they can attract specific metal ions. This section presents the results of structural analysis for both the unmodified sorbent (D301) and its thiourea-functionalized version (TD301), which were obtained using techniques like Fourier-transform infrared spectroscopy (FT-IR), scanning electron microscopy (SEM), energy-dispersive X-ray spectroscopy (EDX), X-ray photoelectron spectroscopy (XPS), and zeta potential measurements.

#### 2.1.1. FT-IR Spectroscopic Analysis

**Figure 1:**
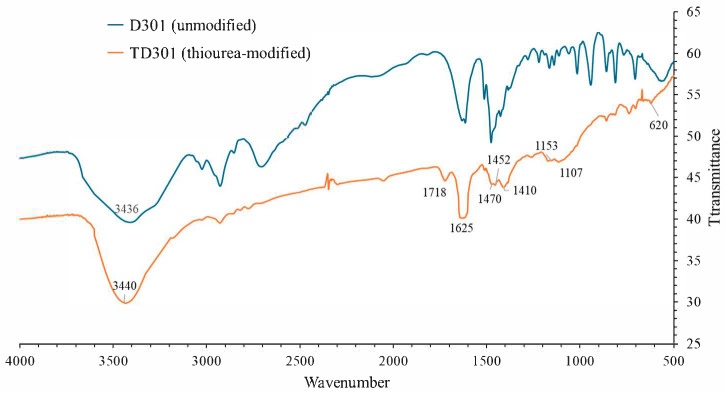
FT-IR spectra of unmodified (D301) and thiourea-modified (TD301) resins.

FT-IR spectra shown in Figure 1 were taken between 4000 and 400 cm^−1^ to find the surface functional groups on D301 and TD301. The blue regions highlight the preserved functional groups of the polystyrene–divinylbenzene matrix. The orange regions correspond to newly introduced functional groups such as amine, hydroxyl, and thiocarbonyl groups. The characteristic stretching vibrations are labeled accordingly.

For the unmodified resin D301, specific absorption bands were observed at 3434 cm^−1^ (O–H/N–H stretching), 2927 cm^−1^ (C–H stretching in aliphatic chains), 1615 cm^−1^ (C=C aromatic ring stretching), and 1016–561 cm^−1^ (C–N, C–H, and skeletal vibrations). These bands correspond to the presence of tertiary amine groups and the polystyrene-divinylbenzene matrix.

After modification, the TD301 spectrum retained these major bands but exhibited several new absorption features. A broadened band at 3440 cm^−1^ confirmed the introduction of additional N–H and O–H functionalities, likely originating from thiourea and hydroxyl groups formed upon ring-opening of glycidyl methacrylate (GMA). Fundamentally, the disappearance of the epoxide absorption around 910 cm^−1^ further supports the occurrence of the ring-opening reaction. An absorption band at 1625 cm^−1^ can be attributed to C=S stretching vibrations while involving contributions from δ(N–H) and C=N vibrations shown in the spectra. This result provides direct evidence of thiourea incorporation. Enhanced bands at 1153 and 1107 cm^−1^ correspond to C–N, C–O, and C=S stretching vibrations, with additional contribution from C–S bonds. Deformation vibrations at 1470, 1452, and 1410 cm^−1^ are associated with newly introduced –CH_2_– and –NH– groups, as well as C–N vibrations typical of thiourea, according to the spectra (NIST database). It can be noted that the weak absorption at 620 cm^−1^ can also be attributed to C–S stretching.

Regarding the coordination of Mo and W species, previous studies indicate that under acidic conditions molybdenum and tungsten occur predominantly as molybdate and tungstate oxoanions (e.g., MoO_4_^2−^, Mo_7_O_24_^6−^, HMoO_4_^−^, WO_4_^2−^, and HWO_4_^−^). The thiourea functional groups (–C=S, –NH–) introduced into TD301 are expected to interact with these species via coordination of the metal centers to the sulfur and nitrogen donor atoms, forming stable complexes. This interaction offers the chemical basis for TD301’s higher affinity for Mo(VI) and W(VI) compared to the unmodified D301 resin. Thus, the structure of the original polymer matrix was preserved, while thiourea-derived functional groups capable of forming complexes with metal ions were successfully introduced.

#### 2.1.2. Scanning Electron Microscopy and Elemental Analysis

**Figure 2:**
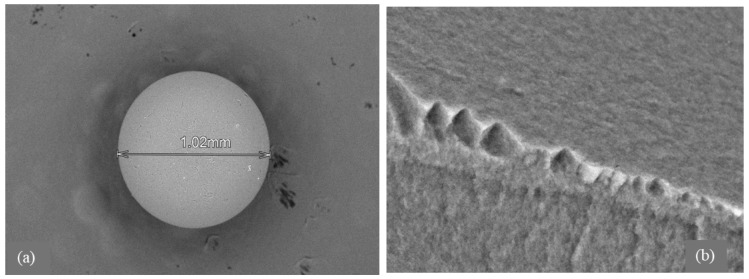

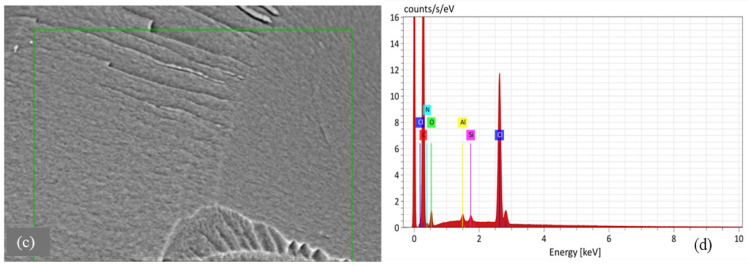
SEM and EDX analysis of unmodified D301 resin: (**a**) Bead morphology; (**b**) Macroporous surface; (**c**) EDX region; (**d**) EDX spectrum.

**Figure 3:**
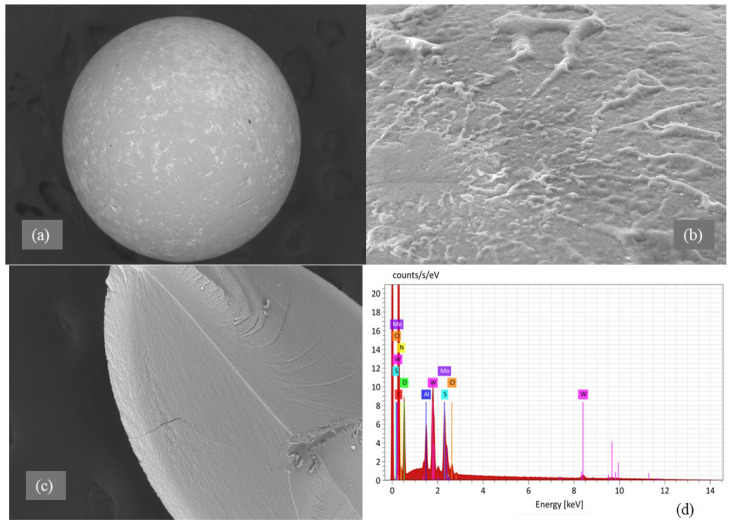
SEM and EDX analysis of thiourea-functionalized resin TD301: (**a**) Bead morphology; (**b**) Functionalized surface; (**c**) EDX region; (**d**) EDX spectrum.

Morphological analysis by scanning electron microscopy (SEM) was provided to both sorbents D301 (Figure 2) and TD301 (Figure 3). It shows a clear porous structure with evenly spread pores (Figure 2b), which matches their ability to swell and their specific volume. The SEM images of the whole resin granules (Figure 2a and Figure 3a) show round particles that are about 1 mm wide, which matches what the manufacturer said. The surface of the modified TD301 resin (Figure 3b) appears smoother due to the addition of grafting and thiourea.

Elemental composition was determined using energy-dispersive X-ray spectroscopy (EDS), with the corresponding SEM micrographs and elemental maps presented in Figure 2c,d and Figure 3c,d. Quantitative results are shown in Table 1 and include the amounts of the detected elements and their possible errors, which were figured out based on how accurately the peaks were measured and how well the instrument was set up. Absolute errors were calculated using the instrument software with standard ZAF correction and correspond to a 1σ confidence level. All reported values represent the average of measurements performed on three distinct surface areas of each sample.

After modification, the TD301 sample showed a significant increase in nitrogen (8.18 ± 0.48 wt.%) and oxygen (19.28 ± 0.79 wt.%), and sulfur (0.57 ± 0.04 wt.%), which confirms that thiourea was successfully functionalized. Sorption experiments showed that molybdenum (8.06 ± 0.55 wt.%) and tungsten (5.06 ± 0.38 wt.%) were present on the surface of TD301, which means it effectively binds heavy metal ions.

The results of FTIR and SEM analyses are mutually supportive. The FTIR spectra indicate the presence of functional groups (–OH, –NH, –CH_2_, –C–S), characteristic of weakly basic ion exchangers, while SEM analysis reveals a well-developed porous structure and high carbon content, consistent with the organic nature of the sorbent. The presence of chlorine, detected by EDX, confirms the existence of ion-exchange groups as established by FTIR.

The studies on the D301 sorbent show that it has a well-developed porous structure and functional groups that enhance its ability to exchange ions.

#### 2.1.3. Zeta Potential Analysis

**Figure 4:**
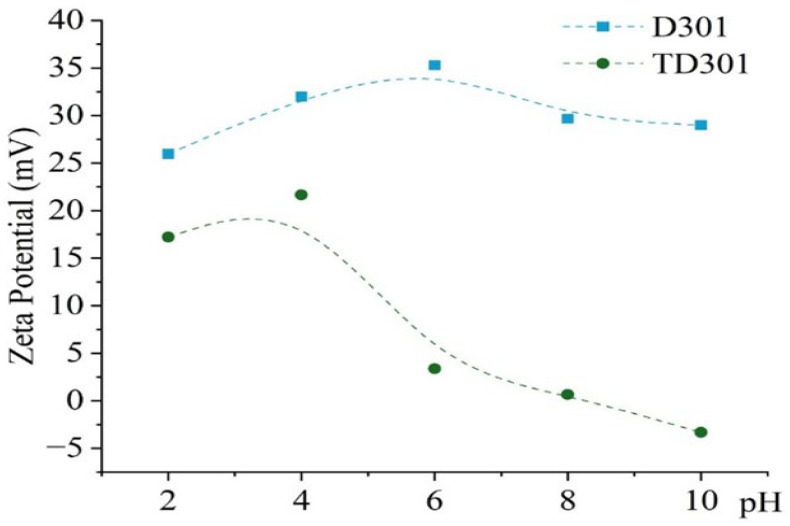
Zeta potential of modified (TD301) and unmodified (D301) sorbents.

Figure 4 presents the zeta potential dependence of the unmodified and thiourea-modified sorbents on pH. All measurements were conducted in aqueous medium at 298 K. The pH range was extended up to 10 not only to cover the working interval of the sorbents but also to establish whether the isoelectric point (IEP) could be reached and to confirm the electrokinetic stability of the surface. Although sorption experiments were carried out only up to pH 6, the extended measurement range provides a complete electrokinetic profile and demonstrates that the modification remains stable even under non-sorption conditions.

The unmodified resin (D301) showed a steady positive surface charge across all pH levels from 2 to 10, with zeta potential values starting at +25.9 mV at pH 2, increasing to +35.3 mV at pH 6, and then slightly dropping to +29.0 mV at pH 10. This shift towards negative or neutral values confirms the substitution of strongly cationic amine/chloride groups by thiourea-derived moieties. These sulfur-containing and nitrogen-containing functionalities are less ionically dominant, and they can also act as donor ligands, which reduces the overall electrostatic contribution and favors specific coordination or chelation with metal ions.

Although HCl was used during the pre-treatment step, its competitive effect at alkaline pH values is not relevant for the present study, since sorption experiments were restricted to acidic and near-neutral conditions (pH ≤ 6).

In contrast, the modified sorbent (TD301) demonstrated a markedly different surface behavior. The zeta potential decreased progressively from +17.2 mV at pH 2 to +0.6 mV at pH 8 and eventually became negative (−3.3 mV) at pH 10. The observed trend is attributed to the introduction of thiourea fragments via graft polymerization, which resulted in the formation of sulfur- and nitrogen-containing functional groups on the surface. Unlike strongly cationic amine/chloride groups, these moieties are neutral or slightly acidic and therefore lower the overall positive surface charge. More importantly, they provide donor atoms capable of specific coordination or chelation with metal ions, which explains the gradual transition from positive to nearly neutral or negative zeta potential values with increasing pH.

The big decrease in zeta potential of TD301 compared to D301 indicates that there are fewer purely electrostatic interactions and more specific binding methods (like chelation or complexation) happening when metal ions are being absorbed. The near-zero surface charge observed at pH 6–8 is particularly important, since it minimizes electrostatic repulsion with anionic species and enhances metal coordination through donor atoms introduced during modification.

Overall, these results demonstrate that surface modification with thiourea not only provides functional groups capable of complexation but also changes the electrokinetic characteristics of the sorbent in a regulated manner, thus boosting its selectivity and adaptability under the studied pH settings.

#### 2.1.4. X-Ray Photoelectron Spectroscopic Analysis

**Figure 5:**
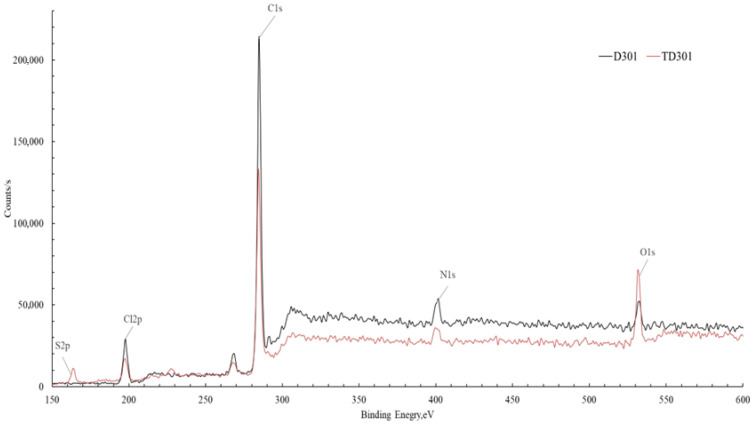
Comparative XPS spectra of the unmodified sorbent D301 and the modified sorbent TD301.

A survey XPS analysis was performed to enhance the reliability of the surface modification, which was confirmed accurately, and this analysis allowed for the assessment of changes in the surface elemental composition of the sorbents resulting from the modification. Figure 5 presents a comparison of the spectrum of the unmodified sorbent D301 and the modified sorbent TD301. Since the samples analyzed are non-conductive, peak shifts caused by surface charging were observed. To obtain accurate data, we adjusted the measurements by using the carbon peak as a reference, shifting the spectrum by −1.3 eV on the energy scale.

Both lines of the spectra showed signals corresponding to C1s (~285 eV), O1s (~532 eV), N1s (~400 eV), S2p (~168 eV), and Cl2p (~200 eV) were identified [34,35]. A noticeable rise in the strength of the O1s and S2p signals in the spectrum of the modified material shows that oxygen- and sulfur-containing functional groups have been successfully added to the sorbent surface.

The summary of the quantitative elemental analysis is presented in Table 2, reflecting the changes in surface composition induced by the modification process.

The results from the quantitative analysis (Table 2) show important changes in the surface elements of the sorbent after it was modified. The most pronounced is the decrease in carbon content (from 79.00 to 56.15 at.%), accompanied by an increase in oxygen, nitrogen, sulfur, and chlorine concentration elements associated with the introduced functional groups.

The increase in oxygen content (from 2.79 to 7.13 at.%) confirms the successful immobilization of poly(glycidyl methacrylate) (PGMA) on the sorbent surface, which contains oxygen-containing groups (–C–O–, –C=O). The increased nitrogen content (from 5.29 to 9.52 at.%) and the appearance of sulfur (5.90 at.%), which was completely absent in the initial sorbent, provide direct evidence of chemical modification with thiourea. This increase confirms the formation of characteristic functional groups, such as amino (–NH_2_) and thiocarbonyl (–C=S) groups, which contribute to the binding of metal ions on the surface of the modified sorbent.

In addition, the higher chlorine content (from 12.89 to 21.30 at.%) shows that the ion-exchange groups of the sorbent were successfully fixed during acid activation. This may enhance the sorption capacity of the material by increasing the number of active ion-exchange sites.

The successful modification of D301 was confirmed by FT-IR, SEM/EDX, zeta potential, and XPS analyses. FT-IR spectra showed the introduction of thiourea-derived groups (C=S, C–N, –NH), while SEM revealed increased surface roughness due to grafted polymer layers. EDX and XPS analyses confirmed the presence of sulfur and elevated levels of nitrogen and oxygen, indicating successful thiourea immobilization. Zeta potential measurements showed a shift toward neutral/negative values at pH 4–6, suggesting a transition from ion-exchange to coordination-based sorption. These combined results confirm the formation of selective binding sites and improved sorption behavior of TD301 toward Mo(VI).

### 2.2. Sorption Behavior in Monometallic Systems

To evaluate the basic characteristics of molybdenum and tungsten sorption, kinetic and pH-dependent experiments were carried out using both unmodified and modified sorbents in monometallic solutions.

#### 2.2.1. Sorption Kinetics of the Unmodified Sorbent

**Figure 6:**
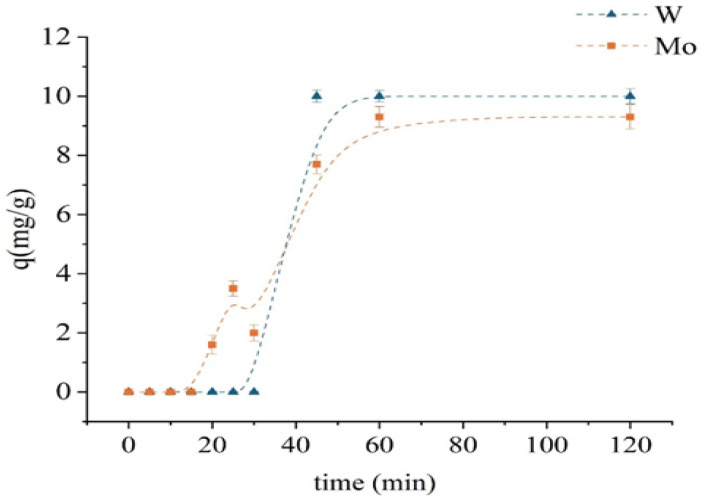
Kinetic sorption curves of monometallic systems using the unmodified D301 sorbent.

Figure 6 presents the kinetics of Mo and W sorption on the unmodified D301 resin. The experiments were carried out with initial concentrations of 100 mg/L for both [Mo^6+^] and [W^6+^], at a solid-to-liquid ratio of 1:100 temperature of 298 K, and a stirring speed of 320 rpm. During the first 20 min, no significant uptake of either molybdenum or tungsten was observed. The sorption of molybdenum gradually increased—from 1.6 mg/g at 20 min to 9.3 mg/g at 60 min—after which it reached saturation. In contrast, tungsten sorption remained low during the first 30 min and then increased sharply, reaching 10 mg/g by the 45th minute and subsequently stabilizing. Such behavior indicates a delay in the initial stage of sorption, which may be attributed to diffusion limitations or the necessity of activation of ion-exchange sites. Equilibrium was attained within 60 min for both metals, and this contact time was selected for subsequent batch experiments. The faster sorption of tungsten is consistent with the high electronegative affinity of the resin for anionic species.

#### 2.2.2. Effect of pH on Sorption with the Unmodified Sorbent

**Figure 7:**
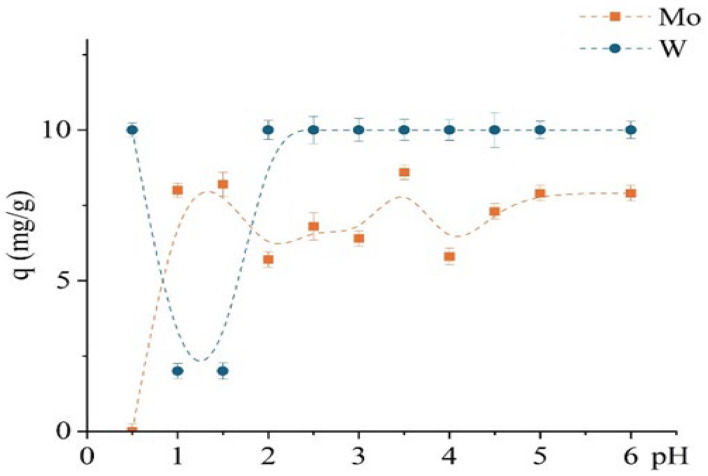
Dependence of the sorption capacity for Mo and W on pH using the unmodified D301 sorbent.

The study investigated the effect of pH (0.5–6) on the sorption performance of the unmodified D301 resin toward Mo(VI) and W(VI), as shown in Figure 7, revealing distinct differences in their uptake behavior. Experimental conditions were similar to those used in the previous kinetic study, with an optimized contact time of 1 h. Molybdenum exhibited a strong pH dependence: sorption increased sharply from pH 1 to 2 and reached a maximum at approximately pH 3.5 (~8.6 mg/g), followed by a slight decline. In contrast, the sorption of W(VI) was almost independent of pH: complete uptake (10 mg/g) was observed even at pH 0.5 and remained constant up to pH 6.

This suggests that tungsten sorption is governed primarily by electrostatic interactions, consistent with its anionic form in solution. The D301 resin, as a strong-base anion exchanger with a positively charged surface at low to neutral pH values (ζ-potential exceeding +30 mV), favors the retention of anionic tungsten species almost regardless of solution pH.

In contrast, the behavior of Mo(VI) is more complex due to its existence in various protonated forms depending on pH—from cationic (MoO_2_^2+^) and neutral (H_2_MoO_4_) species at low pH to anionic forms at pH values above 3. Thus, molybdenum sorption is limited at very low pH values due to the absence of a negative charge and increases with deprotonation, enabling interaction with the D301 matrix—presumably via weak anion exchange or surface adsorption.

#### 2.2.3. Sorption Using the Modified Sorbent TD301

**Figure 8:**
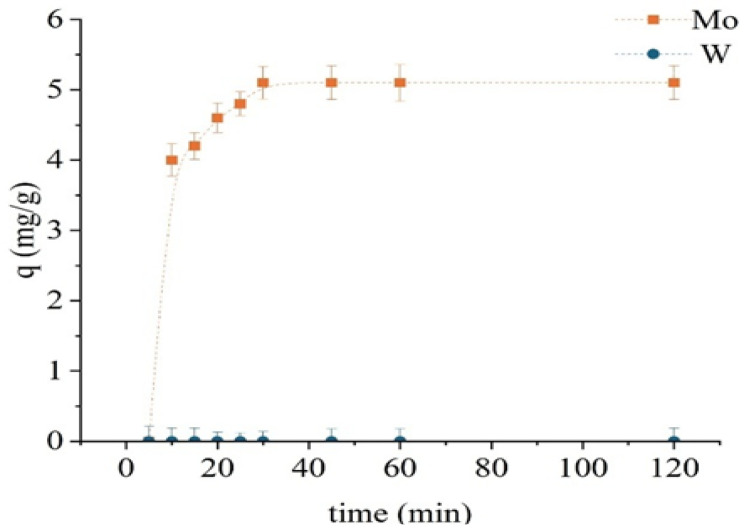
Sorption kinetics of Mo and W using the modified sorbent TD301.

Following chemical modification, the TD301 sorbent exhibited enhanced selectivity and faster sorption kinetics (Figure 8). The experimental setup was consistent with that used in previous tests. Exceptionally rapid molybdenum uptake was observed, with sorption capacity reaching 5.1 mg/g within just 30 min. No further increase was detected beyond this point, indicating rapid saturation. Tungsten was not sorbed at any stage, underscoring the high selectivity of the modified sorbent for molybdenum. This suggests the formation of selective chelating centers on the sorbent surface.

Accordingly, the optimal contact time for TD301 was established as 30 min. The obtained results demonstrate that surface functionalization reduces the required sorption time and significantly enhances the preference for Mo (VI), likely due to chelation involving ligands with sulfur and nitrogen donor atoms.

**Figure 9:**
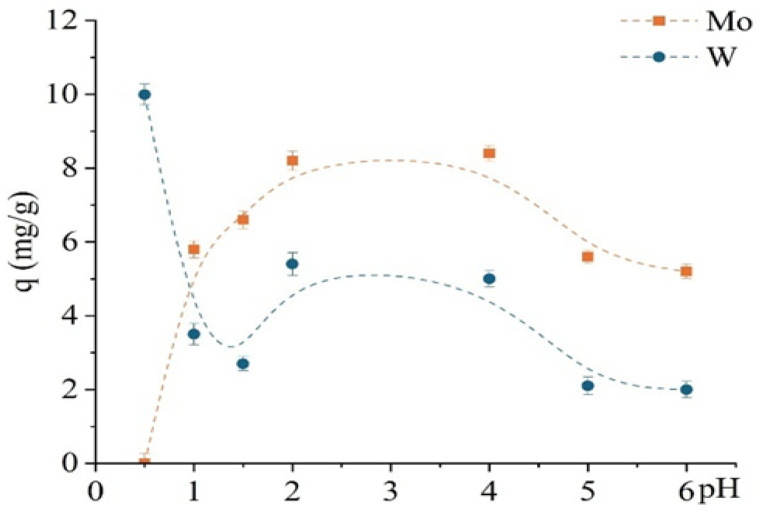
pH dependence of sorption capacity for Mo and W using the modified sorbent TD301.

Figure 9 shows that TD301 effectively targets molybdenum in a wide pH range (1–6), unlike the unmodified D301 sorbent. However, at pH 0.5, preferential sorption of tungsten is again observed. Experimental conditions were similar to those used in prior sorption studies, except for a reduced contact time of 30 min. This effect can be attributed to electrostatic attraction of tungsten anions, while protonated functional groups on the TD301 surface hinder chelation of molybdenum.

The pH-dependent sorption profile of TD301 is markedly different and reveals improved selectivity. Molybdenum sorption significantly increases, reaching a maximum at pH 4 (~8.4 mg/g), whereas tungsten sorption is almost entirely suppressed at pH ≥ 2. Only at pH 0.5–1.5 is minor tungsten uptake observed, which remains considerably lower than that of molybdenum.

This pronounced selectivity for molybdenum is attributed to the successful incorporation of thiourea-based functional groups into the sorbent structure, as confirmed by FTIR (appearance of C=S and NH_2_ bands) and SEM/EDX analysis (presence of sulfur and nitrogen). These donor atoms help to bind Mo(VI)—which is a softer Lewis acid—more effectively than W(VI), because W(VI) is more attracted to water and less likely to form bonds with sulfur and nitrogen atoms.

Additionally, zeta potential measurements indicated that the surface charge of TD301 becomes nearly neutral in the pH range of 3–5. This reduces nonspecific ionic attraction and promotes the formation of specific chelating bonds, which dominate at higher pH. At low pH (0.5–1.5), the addition of protons to donor groups weakens their ability to bond, which allows some tungsten to be absorbed through electrostatic interactions.

### 2.3. Sorption from Binary Mo–W Systems

To simulate real processing conditions and evaluate the selectivity of the sorbents, sorption experiments were carried out using binary Mo–W solutions with varying Mo:W molar ratios (from 1:5 to 5:1) at two pH values: 0.5 and 1.5. The concentrations of each metal ranged from 100 to 500 mg/L, and experiments were conducted at 298 K with a solid-to-liquid ratio (S:L) of 1:100 and a contact time of 30 min under constant stirring (320 rpm). The calculated separation factors *α*(Mo/W) and *α*(W/Mo), which reflect the selectivity toward molybdenum and tungsten, respectively, are presented in Table 3. In this work, the selectivity coefficient was defined as *α* = *D*(Mo)/*D*(W), where *D* is the distribution ratio. Values corresponding to concentrations below LOD were censored; therefore, instead of “∞”, lower boundary estimates are reported (e.g., *α*(W/Mo) > 4.72 × 10^4^). Values denoted as “>4.72 × 10^4^” indicate that the separation factor exceeded the reliable calculation range. This occurred when the residual concentration of one of the ions was below the detection limit, leading to an overestimation of α. This approach avoids unrealistic values and more accurately reflects experimental limitations. In the table, the notation ‘n/a’ indicates that neither metal was sorbed under the given conditions; ‘—’ represents data excluded due to possible experimental error or contamination.

Under strongly acidic conditions (pH = 0.5), the sorption of tungsten consistently exceeded that of molybdenum across all Mo:W ratios for both unmodified (D301) and modified (TD301) sorbents. This trend was particularly evident in equimolar and tungsten-rich systems, where *α*(Mo/W) ≈ 0 and *α*(W/Mo) → >4.72 × 10^4^, indicating complete absence of molybdenum sorption and efficient extraction of tungsten.

In equimolar mixtures (1:1) and tungsten-enriched systems (1:2 to 1:5), Mo(VI) predominantly exists in neutral or weakly cationic forms, which experience little to no electrostatic attraction to the positively charged surface of the resin.

In contrast, W(VI) exists as negatively charged polyoxometalates that are strongly attracted to the positively charged surface of both D301 and TD301, as shown by zeta potential measurements indicating values between +17 and +26 mV at pH 0.5.

Even in molybdenum-rich systems (e.g., 4:1 and 5:1), tungsten maintained preferential sorption, although *α*(W/Mo) values slightly decreased, indicating partial competition at higher Mo concentrations. This emphasizes that at pH 0.5, electrostatic interactions dominate and do not favor molybdenum separation, regardless of the sorbent modification.

Sorption behavior at pH = 1.5 revealed significant differences between the two sorbents:

D301 (unmodified): In equimolar systems (1:1) and Mo-enriched solutions (3:1 and 5:1), effective separation was not achieved; post-sorption concentrations of Mo and W were either equal to or even exceeded initial values. These anomalies may indicate the formation of Mo–W heteropolyionic complexes, which hinder the differentiation of individual metal species and reduce sorption selectivity. In W-enriched systems (1:3, 1:4), both metals were sorbed simultaneously, suggesting nonspecific competition for active sites. Selective sorption of molybdenum was observed only at Mo:W ratios of 2:1 and 4:1, with *α*(Mo/W) values of 1.30 and 1.16, respectively. This suggests that in moderately Mo-enriched solutions, D301 is capable of limited ion discrimination, likely due to weak exchange interactions.

TD301 (modified): The modified sorbent demonstrated significantly improved selectivity for Mo(VI) in most binary systems—with *α*(Mo/W) > 1 in almost all cases, except for 1:5, 3:1, and 4:1. In Mo-enriched systems (e.g., 5:1 and 2:1), molybdenum was selectively sorbed, while tungsten was virtually unretained. However, in tungsten-excess mixtures (1:5), the high content of tungstate likely inhibited Mo binding, thereby reducing selectivity. In intermediate ratios (3:1 and 4:1), unexpectedly weak selectivity was observed (*α* < 1 or “—”), possibly due to the formation of bimetallic complexes that block access to active sites or alter coordination dynamics.

These results indicate that chelating interactions on TD301 are sensitive to metal speciation and competitive effects. When there is too much tungstate, its strong negative charge might push Mo out or fill up the binding spots through weak, non-specific connections, especially when polyoxometalate clusters are also present.

Sorbent behavior across all ratios and pH values confirms the following mechanisms: At pH = 0.5, surface charge plays the primary role: W sorption is driven by electrostatics, while Mo is not retained due to lack of negative charge. At pH 1.5, some thiourea groups on TD301 undergo partial deprotonation, allowing coordination with Mo(VI) through sulfur and nitrogen donor atoms. Compared with W(VI), Mo(VI) exhibits a stronger affinity toward sulfur, leading to improved TD301 selectivity. This behavior contrasts with D301 and D301-g-PGMA, where sorption occurs mainly via electrostatic interactions with limited selectivity. The enhanced performance of TD301 is therefore attributed to the additional coordination mechanism introduced by thiourea groups. The unusual results seen at Mo:W ratios of 3:1 and 4:1 are probably caused by the formation of mixed metal complexes and need more study using molecular modeling and spectroscopic techniques. The modified TD301 sorbent works very well to separate Mo(VI) and W(VI) in acidic conditions, especially at pH = 1.5 and when there is a lot of Mo present. However, its selectivity may decline in specific intermediate compositions, likely due to mutual interactions between the metals.

### 2.4. Separation of Mo and W from Real Leachates

To test how well the developed sorbents work in real life, experiments were performed with an actual ore sample taken from a geologically active area in Central Kazakhstan. The sample represented a typical mineralized material containing Mo and W, currently at the exploration stage.

Ore decomposition was carried out using a mixture of mineral acids (HCl, HNO_3_, and H_2_SO_4_), resulting in a clear solution containing molybdenum and tungsten at equimolar concentrations (~400 mg/L of each metal). The solution also contained accompanying elements such as silicon, iron, and aluminum. Silicon was removed by filtration, as it precipitated during the decomposition process. Iron was precipitated with ammonia following standard procedures. Aluminum, present in low concentrations, did not interfere with Mo and W sorption in acidic media. The final solution had an acidity of approximately pH 0.66, which is close to the previously investigated model conditions (pH 0.5).

The separation efficiency was tested at two acidity levels: 0.66 (the natural acidity of the leachate) and 1.5 (adjusted), using both unmodified D301 and modified TD301 sorbents. The experiments were performed with equimolar concentrations of Mo and W (~400 mg/L each) at 298 K, using a solid-to-liquid ratio of 1:100 and a contact time of 30 min. The resulting separation factors *α*(Mo/W) and *α*(W/Mo) are summarized in Table 4.

At pH 0.66, both sorbents exhibited selective sorption of tungsten, while molybdenum was not sorbed at all. This behavior is consistent with the previously obtained results in model systems at pH 0.5. The high positive surface charge on both sorbents in these very acidic conditions helps attract negatively charged tungstate ions, but Mo(VI), which is likely in a positive form, is not held onto. TD301 demonstrated a significantly higher sorption capacity for tungsten, reflected in a markedly greater separation factor (*α*(W/Mo) > 16 000), which proves that the surface modification worked well.

At pH 1.5, an opposite trend was observed: molybdenum was predominantly sorbed, while tungsten was almost completely excluded. The separation factor *α*(Mo/W) increased to 3.82 for TD301 and to 2.53 for D301, showing that the modified sorbent is better at attracting molybdenum in slightly acidic conditions. This improvement correlates with the activation of thiourea-based chelating centers, which coordinate Mo(VI) more effectively than W(VI).

Thus, the obtained results confirm the validity of laboratory observations under real conditions and demonstrate the effectiveness of TD301 for separating molybdenum and tungsten in complex matrices. At the natural acidity of the ore leachate (pH ≈ 0.66), it is possible to selectively extract tungsten because of beneficial effects from the surface charge. At a mildly acidic pH of 1.5, it is possible to selectively extract molybdenum, particularly with TD301, because the functional groups become more active and there are fewer unwanted electrostatic interactions. These results show that by changing the pH, we can selectively recover either molybdenum or tungsten from solutions made during the processing of natural ores.

## 3. Materials and Methods

### 3.1. Chemicals

The starting sorbent used was the anion-exchange resin D301, purchased from Langfang Senate Chemical Co., Ltd. (Langfang, Hebei, China). The resin was modified using the following chemicals: glycidyl methacrylate (C_7_H_10_O_3_, purity ≥ 99.5%) from Qianjin Reagent (Hangzhou, Zhejiang, China); N,N-dimethylformamide (purity ≥ 99.8%) and ammonium persulfate ((NH_4_)_2_S_2_O_8,_ ≥98.0%) from Sigma-Aldrich (St. Louis, MO, USA); thiourea (CH_4_N_2_S, ≥99.0%) from Beijing Chemical Plant (Beijing, China); and acetone (C_3_H_6_O, ≥99.0%) from AppliChem GmbH (Darmstadt, Germany). All chemicals were used without further purification.

Ammonium tungstate ((NH_4_)_10_H_2_(W_2_O_7_)_6_, purity ≥ 99.9%) and ammonium molybdate ((NH_4_)_2_MoO_4_, ≥99.98%), purchased from Sigma-Aldrich, were used to prepare standard solutions of the metal ions under investigation.

Sodium hydroxide (NaOH, ≥98.0%) was supplied by Kazkhimtehsnab LLP (Semey, Kazakhstan), and hydrochloric acid (HCl, ≥37.0%) was supplied by ECOS-1 (Almaty, Kazakhstan). All inorganic reagents were of analytical grade and were used without further purification. Deionized water was used throughout all stages of solution preparation and experimental procedures.

### 3.2. The Method for Preparing a Modified Styrene-Based Sorbent for Sorption of Molybdenum and Tungsten

**Figure 10:**
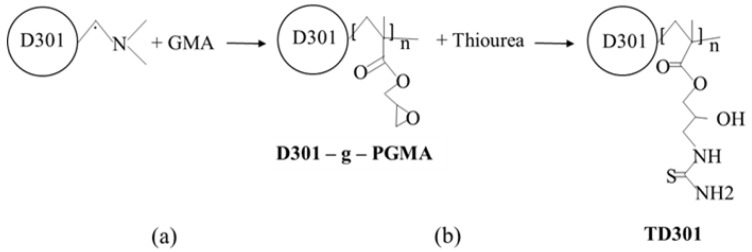
Preparation process of thiourea-functionalized resin TD301: (**a**) Graft Polymerization; (**b**) Thiourea Functionalization.

To introduce reactive functional groups into the structure of the macroporous anion exchange resin D301, a two-step chemical modification was carried out (Figure 10). This process involved graft polymerization of glycidyl methacrylate (GMA) (Figure 10a) followed by functionalization with thiourea (Figure 10b).

In the first stage, D301 was dispersed in dimethylformamide (DMF) using a ratio of one part D301 to 100 parts DMF. GMA was then added to a sorbent-to-monomer ratio of 1:10. Ammonium persulfate, at 1.5% of the GMA mass, was used as the initiator for radical graft polymerization. The grafting reaction was carried out at 323 K for 6 h under an inert nitrogen atmosphere to prevent oxidative degradation. The resulting modified material was denoted as D301-g-PGMA.

Upon completion of polymerization, particle agglomeration was observed upon exposure to air and moisture, indicating the presence of physically adsorbed or loosely bound polymer chains. To remove these fragments, the product was treated in a Soxhlet apparatus using acetone, which effectively removed loosely attached residues without harming the grafted layer.

In the second stage, the grafted sorbent was functionalized with thiourea in an alkaline aqueous medium. The reaction was carried out in a sodium hydroxide solution with a concentration of 1 × 10^−4^ M, using 1 part D301-g-PGMA to 15 parts thiourea by weight. The modification was performed at 343 K for 8 h. After the reaction, the product was washed several times with acetone in a Soxhlet apparatus, then with deionized water, dried, and labeled as TD301.

Functionalization of the epoxy groups with thiourea resulted in the formation of coordination-active sites containing sulfur and nitrogen atoms, which exhibit a high affinity for heavy metal ions. This makes the resulting material a promising sorbent for the removal of toxic metals such as Mo(VI) and W(VI) from aqueous media through mechanisms of ion exchange and complexation.

### 3.3. Pre-Treatment Before Sorption

Prior to sorption experiments, the modified sorbent was activated by immersion in 1 mol/L hydrochloric acid. This treatment helped to protonate functional groups and remove residual impurities. The resin was then rinsed repeatedly with deionized water until a neutral pH was achieved. This pre-treatment ensured the reproducibility of sorption behavior and stability of surface chemistry.

### 3.4. Characterization of Initial and Modified Macroporous Anion-Exchange Resin

To evaluate the physical and chemical properties of the initial and modified weak-base macroporous anion-exchange resin, a series of characterization techniques were used.

Fourier Transform Infrared Spectroscopy (FTIR) was conducted using a PerkinElmer Spectrum 65 FT-IR spectrophotometer (Shelton, CT, USA) within the spectral range of 400–4000 cm^−1^ to identify the functional groups present on the resin surface and to confirm the chemical modifications.

The surface morphology and structural features of the sorbent before and after modification were examined using a Hitachi TM4000 scanning electron microscope (SEM) (Tokyo, Japan). Imaging was performed at an accelerating voltage of 15 kV, with working distances between 7.4 and 8.1 mm, and at magnifications ranging from ×50 to ×1000. Both secondary electron (SE) and backscattered electron (BSE) detectors were used to give additional details about the surface shape and material differences. Elemental composition was analyzed using a Bruker XFlash 6/30 energy-dispersive X-ray spectrometer (EDS) (Karlsruhe, Germany) at 400× magnification. These analytical conditions enabled detailed characterization of surface texture and porosity, which are critical factors influencing sorption performance.

Zeta potential measurements were conducted to assess the surface charge characteristics of the sorbent as a function of pH, which is a critical parameter for understanding the interaction between the sorbent and anionic species. The evaluation of surface charge is important for understanding how stable the particles are and how they attract certain ions, which helps determine how well the sorbent works at different pH levels.

All measurements were performed using a Zetasizer Advance Series (Malvern Light Scattering Technology, ZSU3200-Pro, Malvern Panalytical, Malvern, UK) at a temperature of 298 K. The samples were prepared by dispersing the sorbent in an aqueous medium and adjusting the pH using 0.1 M HCl or NaOH. The pH range was selected to match the usual conditions for the sorbent and to prevent metal hydroxides from forming, as they could affect the accuracy of the zeta potential measurements.

The zeta potential values were calculated using Stokes’ equation (Equation (1)), which is suitable for colloidal systems where the thickness of the double electric layer is significantly smaller than the particle size. This condition is typical for the synthesized sorbent particles due to their fine dispersion and low ionic strength in the medium. The equation used is as follows:

(1) $$\zeta =\frac{4\pi \eta ս}{\varepsilon \varepsilon_{0}}$$

where *ζ* (V) is the zeta potential, *η* (Pa·s) is the dynamic viscosity of the medium, *ս* (m^2^/(V·s)) is the electrophoretic mobility, *ε* (dimensionless) is the relative permittivity of the medium, and *ε*_0_ (8.854 × 10^−12^ F/m) is the permittivity of free space.

The choice of Stokes’ equation was justified based on the colloidal nature of the sorbent particles, where the thickness of the electrical double layer is negligible compared to the particle size, allowing the equation to accurately describe the zeta potential in low ionic strength conditions. The results obtained from the zeta potential analysis were used to interpret the electrostatic interaction behavior of the sorbent, particularly its affinity toward anionic species.

To evaluate the surface elemental composition of the ion-exchange sorbents before and after modification, X-ray photoelectron spectroscopy (XPS) was performed using a Thermo Scientific Nexsa instrument (Waltham, MA, USA). Two samples were analyzed: the unmodified sorbent D301 and the modified sorbent TD301. Both sorbents were pre-activated in 1 M hydrochloric acid solution, then dried and ground. Based on the obtained spectra, the areas of the characteristic peaks will be calculated and normalized using sensitivity factors, and the relative atomic concentrations of the elements will be determined.

### 3.5. Adsorption Experiments

Batch sorption experiments were carried out using a fixed sorbent-to-solution ratio of 10 g of sorbent per liter of solution (10 g/L) under constant agitation at 320 rpm and a controlled temperature of 298 K. The experiments were performed using a Premium Hotplate Stirrer SMHS-6 (Daihan Scientific Co., Ltd., Wonju, Republic of Korea). Upon completion of the sorption process, the solid and liquid phases were separated by filtration. The residual concentrations of metal ions in the solutions were determined using inductively coupled plasma mass spectrometry (ICP-MS, Agilent 7500a, Santa Clara, CA, USA). All experiments were carried out in triplicate, and the results were expressed as the mean values of three parallel measurements.

The effects of contact time, initial metal concentration, solution pH, metal ion ratios on the sorption behavior and separation efficiency were systematically investigated. The sorption capacity and removal efficiency were calculated according to Equations (2) and (3) as follows:

(2) $$q=\frac{\left(C_{0}-C_{i}\right)\times V}{m}$$

(3) $$E=\frac{\left(C_{0}-C_{i}\right)}{C_{0}}\cdot 100\%$$

where *q* (mg/g) is the sorption capacity, *E* (%) is the sorption efficiency, *C*_0_ and *C_i_* (mg/L) are the initial and final metal ion concentrations, respectively, *V* (L) is the volume of the solution, and *m* (g) is the mass of the sorbent.

### 3.6. Sorption from Binary Systems

To evaluate the separation efficiency of Mo and W from binary systems, a series of solutions with varying molar ratios of Mo to W were prepared. The experimental design included two sets of binary mixtures: one in which Mo predominated and another where W was in excess. Additionally, a solution with equimolar concentrations of both metals was prepared to study how they behave when competing with each other.

The experiments were conducted under the same conditions as for the monometallic systems—with continuous stirring until sorption equilibrium was reached. The residual concentrations of Mo and W after sorption were measured using inductively coupled plasma mass spectrometry (ICP-MS, Agilent 7500a), which gave a trustworthy evaluation of how well the metals were separated when they were competing with each other.

The separation efficiency was measured using the separation factor (*α*), which is the ratio of the distribution coefficients (*D*) of the two metals, as shown in Equations (4) and (5) as follows:

(4) $$\alpha_{W}^{Mo}=\frac{D_{Mo}}{D_{W}}=\frac{{(C}_{0}\left(Mo\right)-C(Mo))/C(Mo)}{{(C}_{0}\left(W\right)-C(W))/C(W)}=\frac{C_{sorb}(Mo)/C_{sol}(Mo)}{C_{sorb}(W)/C_{sol}(W)}$$

(5) $$\alpha_{Mo}^{W}=\frac{D_{W}}{D_{Mo}}=\frac{{(C}_{0}\left(W\right)-C(W))/C(W)}{{(C}_{0}\left(Mo\right)-C(Mo))/C(Mo)}=\frac{C_{sorb}(W)/C_{sol}(W)}{C_{sorb}(Mo)/C_{sol}(Mo)}$$

where *C*_0_ and *C* (mg/L) are the initial and final concentrations of the respective metal ions. These parameters allowed for a quantitative evaluation of the sorbent’s selectivity for molybdenum over tungsten at different concentration ratios.

## 4. Conclusions

In this study, the commercial macroporous anion-exchange resin D301 was modified via graft polymerization of glycidyl methacrylate, followed by functionalization with thiourea. As a result, a novel sorbent, TD301, with enhanced selectivity toward molybdenum(VI) was obtained. Material characterization was carried out using SEM/EDS, FTIR, X-ray photoelectron spectroscopy, and zeta potential analysis, while sorption performance was evaluated in both mono-metal and multicomponent Mo–W systems, including real leachates derived from ore.

SEM/EDS analysis showed that sulfur- and nitrogen-containing groups were successfully added, the surface became rougher, and the active sites were evenly spread out. FTIR revealed the appearance of new absorption bands corresponding to thiourea-based functional groups (C=S, –NH_2_), validating the chemical modification of the resin. Zeta potential measurements indicated that the surface charge of TD301 was lower than that of D301, especially between pH levels 2 and 6, which helps improve specific sorption by reducing unwanted electrostatic interactions. The X-ray photoelectron spectroscopy (XPS) analysis showed important changes in the surface elements of the sorbent after it was modified, with higher amounts of oxygen, nitrogen, sulfur, and chlorine, confirming that functional groups were successfully added. These structural transformations contribute to the formation of active sites responsible for the selective binding of metal ions.

In mono-metal systems, TD301 exhibited rapid sorption kinetics, reaching equilibrium within 30 min (compared to 60 min for D301). At pH 0.5, sorption of tungsten prevailed due to strong electrostatic attraction between the positively charged surface and anionic tungstate species. In contrast, at pH 1.5, TD301 showed satisfactory separation by favoring the absorption of molybdenum, which was due to specific interactions. These pH values were found to be optimal for the selective separation of the two metals.

In binary Mo–W systems, TD301 demonstrated high selectivity for Mo, with a separation factor *α*(Mo/W) of up to 128.4 at pH 1.5, whereas D301 failed to achieve *α* > 1 under similar conditions. Experiments with real ore leachate solutions (Mo ≈ W ≈ 40 mg/L) confirmed the selective extraction of tungsten by TD301 at pH 0.66 (*α*(W/Mo) = 16.139) and of molybdenum at pH 1.5 (*α*(Mo/W) = 3.82).

These findings confirm that thiourea modification of D301 results in the formation of selective chelating sites and enables highly efficient pH-dependent separation of Mo(VI) and W(VI). The dual selectivity mechanism—targeting tungsten at low pH and molybdenum at higher pH—makes TD301 a promising sorbent for the recovery of valuable metals from both industrial and natural solutions.

Moreover, the sorbent can be applied for the monitoring and treatment of wastewater generated during the processing of copper and tungsten–molybdenum ores, as well as other technological solutions containing Mo and W. Its high selectivity, rapid sorption kinetics, and reusability make TD301 a promising material for the implementation of low-waste and sustainable water treatment technologies.

## Figures and Tables

**Table 1:** Elemental composition of D301 and TD301 resins obtained by EDX analysis (with absolute errors, wt.%).

Element	D301 (% wt ± abs. Error)	TD301 (% wt ± abs. Error)	Interpretation
C	78.06 ± 3.96	56.21 ± 2.71	Organic matrix (polystyrene)
N	5.65 ± 0.32	8.18 ± 0.48	Amine groups; increased after thiourea grafting
O	6.60 ± 0.27	19.28 ± 0.79	Hydroxyl/ether groups from PGMA
S	–	0.57 ± 0.04	Sulfur from thiourea
Cl	9.12 ± 0.35	0.65 ± 0.05	Chloride from ion-exchange form
Mo	–	8.06 ± 0.55	Sorbed molybdenum (post-sorption)
W	–	5.06 ± 0.38	Sorbed tungsten (post-sorption)

**Table 2:** Surface elemental composition of unmodified (D301) and modified (TD301) sorbents based on XPS analysis (atomic % ± absolute error).

Element	D301 (at%)	TD301 (at%)
C	79.00 ± 1.2	56.15 ± 1.0
Cl	12.89 ± 0.5	21.30 ± 0.6
N	5.29 ± 0.4	9.52 ± 0.5
O	2.79 ± 0.5	7.13 ± 0.6
S	-	5.90 ± 0.4

**Table 3:** Separation factors (*α*) for Mo/W at various Mo:W molar ratios and pH values using unmodified (D301) and modified (TD301) sorbents.

Mo:W		1:1	1:2	1:3	1:4	1:5	2:1	3:1	4:1	5:1
**pH = 0.5** **D301**	*α*(Mo/W)	1.00 × 10^−3^	n/a	n/a	n/a	n/a	n/a	n/a	0.12	0.09
	*α*(W/Mo)	7.42 × 10^2^	>4.72 × 10^4^	>4.72 × 10^4^	>4.72 × 10^4^	>4.72 × 10^4^	>4.72 × 10^4^	>4.72 × 10^4^	3.18	6.19
**pH = 0.5** **TD301**	*α*(Mo/W)	2.40 × 10^−5^	n/a	n/a	n/a	n/a	n/a	n/a	0.18	0.02
	*α*(W/Mo)	4.18 × 10^4^	>4.72 × 10^4^	>4.72 × 10^4^	>4.72 × 10^4^	>4.72 × 10^4^	>4.72 × 10^4^	>4.72 × 10^4^	5.58	46.89
**pH = 1.5** **D301**	*α*(Mo/W)	−	0.63	4.55 × 10^4^	0.25	0.14	1.13 × 10^4^	−	4.31 × 10^4^	−
	*α*(W/Mo)	−	2.19 × 10^4^	0.14	3.59 × 10^4^	3.59 × 10^4^	0.76	−	0.85	−
**pH = 1.5** **TD301**	*α*(Mo/W)	4.57 × 10^4^	2.05 × 10^4^	17.13	4.38 × 10^4^	-	4.72 × 10^4^	−	−	126.11
	*α*(W/Mo)	0.31	0.39	0.06	0.84	-	0.44	−	−	0.01

**Table 4:** Separation of Mo/W from acidic solution obtained by ore leaching.

Sorbent	pH	*α*(Mo/W)	*α*(W/Mo)
D301	0.66	n/a	5.26 × 10^2^
TD301		n/a	>4.72 × 10^4^
D301	1.5	2.53	0.39
TD301		3.82	0.26
	pH	*α*(Mo/W)	*α*(W/Mo)

## Data Availability

The raw data supporting the conclusions of this article will be made available by the authors on request.
